# LC-HRMS-Based Metabolomic Profiling and Antioxidant Activity of *Sargassum ilicifolium* Under Different Pretreatments, Extraction Methods, and Solvents

**DOI:** 10.3390/antiox15040433

**Published:** 2026-03-31

**Authors:** Anita Dilla Harfiyani, Riyanti, Muhammad Nursid, Till F. Schäberle, Maria Alexandra Patras, Jae-Suk Choi, Maria Dyah Nur Meinita

**Affiliations:** 1 Magister of Agricultural Biotechnology Study Program, Graduate School, Universitas Jenderal Soedirman, Purwokerto 53122, Indonesia; 2 Faculty of Fisheries and Marine Sciences, Universitas Jenderal Soedirman, Purwokerto 53122, Indonesia; 3 Research Center for Food Technology and Processing, National Research and Innovation Agency (BRIN), Playen, Yogyakarta 55861, Indonesia; 4 Faculty of Agricultural Sciences, Nutritional Sciences and Environmental Management, Justus Liebig University, 35390 Giessen, Germany; 5 Natural Product Department, Fraunhofer-Institute for Molecular Biology and Applied Ecology (IME), Ohlebergsweg 12, 35392 Giessen, Germany; 6 Department of Seafood Science and Technology, The Institute of Marine Industry, Gyeongsang National University, 38 Cheondaegukchi-gil, Tongyeong-si 53064, Gyeongsangnam-do, Republic of Korea

**Keywords:** *Sargassum*, LC-HRMS, antioxidant, macroalgae, metabolomic

## Abstract

*Sargassum* is a widespread brown seaweed species and a source of bioactive compounds with promising antioxidant potential. Unfortunately, to date, the *Sargassum* species remains largely unexplored. This study was conducted to explore the bioactive compounds from *Sargassum ilicifolium* extracts collected from Nguyahan and Sundak Beaches, Gunungkidul, Indonesia, by observing Liquid Chromatography–High Resolution Mass Spectrometry (LC-HRMS)-based metabolomics profiling and antioxidant activity assays. Metabolomic analysis detected 506 molecular features across different extraction methods and solvents, with five metabolites putatively dereplicated, including atractylenolide III, pheophorbide A, 13-docosenamide, 1,3,6,8-tetrahydroxy-2-(1-hydroxyhexyl)anthracene-9,10-dione, and 5-hydroxy-6E,8Z,11Z,14Z,17Z-eicosapentaenoic acid. Extraction parameters, particularly solvent polarity and sample pretreatment, have been shown to affect the metabolite variation. Dried samples showed less variation in metabolites than the fresh sample. Antioxidant activity assay showed a moderate to high radical scavenging activity (30–100%), with methanol extracts as a polar solvent inhibited more than semipolar solvents. This study provides a metabolomics-guided assessment of the antioxidant potential of *S. ilicifolium*, supporting its value and potential as a source of bioactive compounds for future pharmaceutical and nutraceutical applications.

## 1. Introduction

The genus *Sargassum* is a group of brown seaweeds that is considered one of the most abundant and widespread marine biomasses in the tropical and subtropical oceans. The Food and Agriculture Organization of the United Nations has reported that the estimated production of aquaculture for the year 2022 is 130.9 million tons globally. This consists of 94.4 million tons for aquatic animals production and 36.5 million tons for algae production, which are mostly dominated by macroalgae or seaweeds [1]. Seaweeds occur both in intertidal areas between the high and low tide zones, and also in subtidal areas down to depths where photosynthetic light is still available. They are classified into three main groups: brown seaweeds (Phaeophyta), green seaweeds (Chlorophyta), and red seaweeds (Rhodophyta). *Sargassum* is a genus of brown seaweed commonly known as gulfweed or sea holly, belonging to the family *Sargassaceae*, order Fucales, subclass Cyclosporeae, and class Phaeophyceae, comprising approximately 400 species [2,3]. *Sargassum* is one of the most important brown seaweeds from an ecological and economic perspective, occurring in the tropical and subtropical oceans, including the Atlantic, Pacific, and Indian Oceans.

The genus *Sargassum* is widely distributed in tropical and subtropical waters, including the southern coastal regions of Java, Indonesia [4]. However, the industrial application of *Sargassum* seaweed is limited in the world due to difficulties in the large-scale processing and development of the value chain of the seaweed. Globally, *Sargassum* species are very abundant but still underutilized. This is a challenge, where the abundance of *Sargassum* species is often left unused or considered as waste, despite their rich biochemical composition and high economic potential. Many studies have shown that brown seaweed contains a variety of secondary metabolites that have the potential as a source of raw materials for functional food [5], cosmetic [6], and pharmacological and nutritional properties [7,8,9,10]. Therefore, exploring the potential of *Sargassum* in Indonesia is crucial to support the sustainable use of marine resources. Among the diverse species in this genus, *Sargassum ilicifolium* is widely distributed in tropical Indo-Pacific coastal waters and is often found in Southeast Asian marine ecosystems, including the Indonesian coast [11]. Metabolomic and pharmacological studies of *Sargassum* have been conducted for *Sargassum duplicatum* [12], while studies on the metabolite composition of *S. ilicifolium* are still very limited. In addition, metabolomic profiling of *S. ilicifolium* from Indonesian coastal waters has not been studied or reported. Therefore, understanding the metabolite composition of *Sargassum* is important to evaluate its biochemical potential. Metabolomics profiling using liquid chromatography-high-resolution mass spectrometry (LC-HRMS) was conducted to analyze metabolites with high sensitivity and resolution [13,14]. This technique allows the detection of diverse compounds with high sensitivity and provides structural information to explore the bioactive chemical diversity of marine macroalgae [12].

This study used *S. ilicifolium* samples, which were collected from two coastal locations with different environmental conditions: Nguyahan Beach and Sundak Beach. The selection of these locations was based on the distribution, abundance, and morphological diversity of *Sargassum* species, as well as the environmental characteristics of these coastal areas [4]. Both beaches are located along the southern coast of Java, which has a dynamic and heterogeneous coastal environment influenced by monsoon forcing and large-scale ocean–atmosphere interactions. Although environmental measurements such as salinity, temperature, and wave exposure for Nguyahan and Sundak Beaches are not yet available, regional studies have reported spatial variability in sea level and hydrodynamic conditions along the southern coast of Java, driven by monsoon winds, El Niño–Southern Oscillation (ENSO), and Indian Ocean Dipole (IOD) phenomenon [15]. Furthermore, variations in sea surface temperature and wind patterns along the southern coast of Java indicate heterogeneous thermal and physical conditions that may affect coastal biota [16]. The karst coastal landscape of Gunungkidul has been shown to play an important role in the release of submarine groundwater into nearshore waters, which may affect coastal water chemistry and nutrient availability [17]. Environmental parameters such as hydrodynamics, temperature, light availability, nutrient input, season, and salinity are known to influence the chemical composition and biological activity of brown seaweeds, including *Sargassum* species [18]. This provides an insight into how environmental variability can influence metabolite composition and antioxidant potential in *Sargassum* populations.

*Sargassum* sp. contains various secondary metabolites responsible for its biological activities, including phenolic compounds, terpenoids, and the pigment fucoxanthin [19,20,21]. These compounds have strong antioxidant properties and provide various health benefits, including antioxidant [22,23,24,25], anticancer [26], antibacterial [27], anti-inflammatory [28], and anti-aging effects [29]. Several studies have reported the bioactive potential of various *Sargassum* species. However, research on different sampling locations, pretreatment, solvents, and extraction methods that may affect the metabolite composition and antioxidant potential of *S. ilicifolium* has not been systematically evaluated. Therefore, this study is needed to address the limitations of metabolite characterization of *S. ilicifolium* in Indonesia. LC–HRMS-based metabolomic profiling was performed on fresh and dry crude extracts, which were obtained using maceration and microwave extraction with three different types of solvents (n-hexane, ethyl acetate, and methanol). The metabolite profiles were then compared with their antioxidant capacity, which was evaluated using a DPPH radical scavenging assay. By correlating pretreatments, extraction methods, solvents, and metabolite composition with antioxidant activity, this study provides insight into how these factors might affect the antioxidant potential of *S. ilicifolium.* This study is also one of the first LC–HRMS-based metabolomics investigations on *S. ilicifolium* collected from Indonesian coastal ecosystems, combining extraction methods, solvents, and antioxidant activity.

## 2. Materials and Methods

### 2.1. Sample Collection and Identification

**Figure 1:**
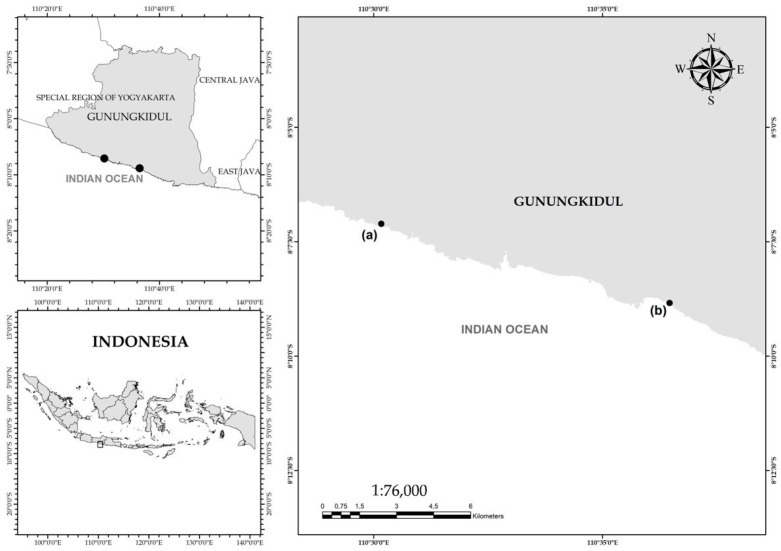
Sampling Location in Gunungkidul, Indonesia. The dots represent the sampling locations at (a) Nguyahan Beach, Gunungkidul, Indonesia and (b) Sundak Beach, Gunungkidul, Indonesia. The grey area indicates landmass of Gunungkidul and the white area indicates the Indian Ocean.

*S. ilicifolium* samples were collected from two different locations, namely Nguyahan Beach (8.118493° S, 110.502771° E) and Sundak Beach (8.147354° S, 110.607767° E), Gunungkidul, Indonesia (Figure 1). The sampling locations were selected based on the abundance of *S. ilicifolium* and the environmental characteristics. A purposive random sampling approach was employed, in which specimens were randomly collected according to their morphological traits [30]. Collected samples were rinsed with sterile seawater to remove debris, epiphytic organisms, and adhering salts, then placed in sterile ziplock bags and stored in an ice box during transport. For metabolomic and antioxidant analyses, samples were subsequently rinsed with freshwater to remove residual impurities. For molecular analyses, young thalli (3–5 cm in length) or approximately 200 g of fresh biomass were selected [31]. The samples were divided into two pretreatment groups: (i) fresh samples, and (ii) dried samples (air-dried and ground into powder prior to analysis). Species identification was initially conducted based on morphological and anatomical characteristics and also confirmed using DNA barcoding.

### 2.2. Metabolomic Profiling

**Figure 2:**
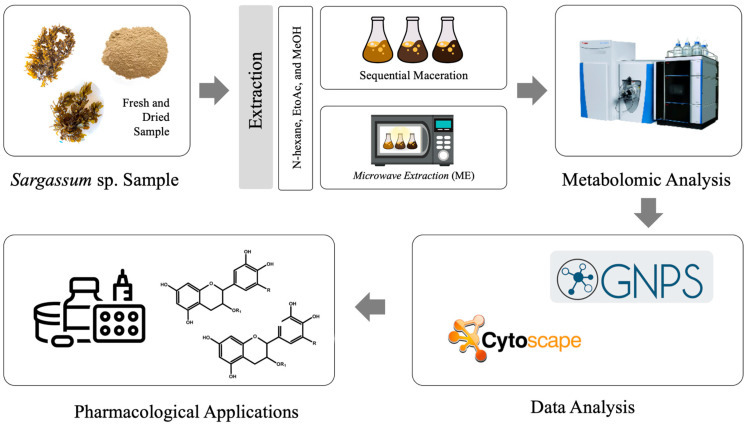
Workflow for LC-HRMS-based metabolomic profiling of *S. ilicifolium*.

**Figure 3:**
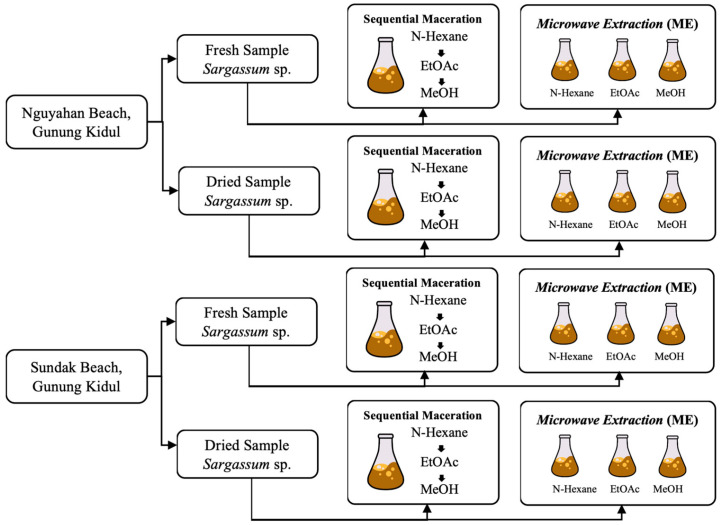
Secondary metabolite extraction of *S. ilicifolium* using maceration and microwave extraction (ME) method.

Metabolites were extracted from fresh and air-dried *S. ilicifolium* samples using sequential maceration and microwave extraction (ME) with three different polarities of solvents (n-hexane, ethyl acetate, and methanol) (Figure 2 and Figure 3). For maceration, samples (1:10 *w*/*v*) were shaken at 180 rpm for 48 h, filtered, and evaporated. The extraction processes were carried out under ambient laboratory conditions, and the temperature was not specifically controlled during extraction. Residues were sequentially extracted with the remaining solvents. For ME, samples (1:10 *w*/*v*) were irradiated for 30 min at medium–high power using a microwave oven (ME-731K, Samsung Electronics, Suwon, Republic of Korea). Extracts were stored at −20 °C until analysis.

LC-HRMS analysis was performed using a micrOTOF-QII mass spectrometer (Bruker Daltonics, Bremen, Germany) with an ESI source connected to an Agilent Infinity 1290 UPLC system with a DAD detector (Agilent, Santa Clara, CA, USA). For the separation, an Acquity UPLC BEH C18 column (2.1 × 100 mm; 1.7 µm; Waters, Eschborn, Germany) was used together with a BEH C18 VanGuard pre-column (2.1 × 5 mm; Waters, Eschborn, Germany). The mobile phase was A (water with 0.1% formic acid) and B (acetonitrile with 0.1% formic acid), with a flow rate of 600 µL/min. The run started at 95% A and kept the same until 0.30 min, then decreased to 4.75% at 18.00 min and reached 0% at 18.10 min. This composition was held until 22.50 min, then followed by re-equilibration to 95% at 22.60 min and kept constant until 25.00 min. The MS data were acquired in the range of 100 to 1500 *m*/*z* in positive mode with an injected volume of 5 μL and a column temperature of 45 °C. 10 mM sodium formate in H_2_O/iPrOH was used as an internal standard for mass spectrum calibration (1:1) [32].

### 2.3. Antioxidant Activity

Antioxidant activity was tested by the 1,1-diphenyl-2-picrylhydrazyl (DPPH) assay. A 1000 μg/mL DPPH stock solution was made in methanol. Ascorbic acid (10 μg/mL) served as a positive control. In a 96-well microplate, 40 μL of the sample was mixed with 120 μL DPPH. Plates were kept in the dark at room temperature for 15 to 30 min. Absorbance at 517 nm was recorded. Percent radical inhibition was calculated for three repetitions.

### 2.4. Data Analysis

Antioxidant activity was calculated as the percentage of inhibition (% inhibition) from three replications and processed using RStudio (version 2025.09.2+418). Prior to further analysis, normality and homogeneity tests were performed using the Shapiro–Wilk and Levene tests. Furthermore, to determine the influence of solvents, a one-way ANOVA test was carried out, followed by a post hoc Tukey’s HSD test. Meanwhile, the combined effect of pretreatment, extraction method, and solvents was analyzed using a three-way ANOVA test. Metabolite compound data were in the form of LC-HRMS data converted to .mzXML format using MS Convert. Data were processed through the Global Natural Products Social Molecular Networking (GNPS) platform, visualized using Cytoscape version 3.10.0. and further spectral analysis was performed using DataAnalysis version 4.0. [32]. Metabolite characterization was analyzed descriptively based on mass measurements and fragmentation patterns of each compound in each sample.

## 3. Results

### 3.1. Metabolomic Profiling

The LC–HRMS-based metabolomic profiling of brown seaweed species, *S. ilicifolium,* showed the diversity of chemical compounds under different pretreatments, various extraction conditions, and different polarities of solvents. Fresh and dried samples collected from Nguyahan and Sundak Beaches, Gunungkidul, Indonesia, were extracted using two different extraction methods, i.e., maceration and microwave extraction (ME). The extraction process was carried out using three solvents with varying degrees of polarity, namely *n*-hexane, ethyl acetate, and methanol.

**Figure 4:**
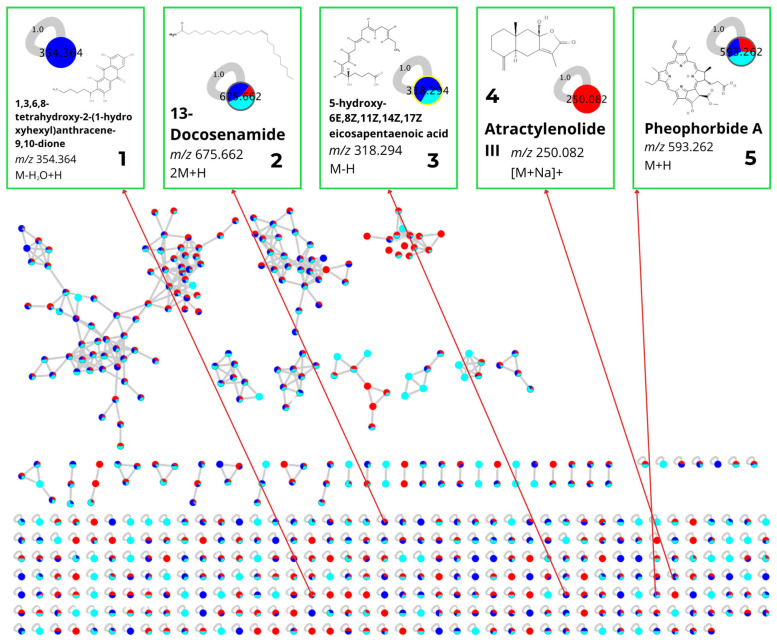
Molecular networking of secondary metabolites from *S. ilicifolium* extracts generated using GNPS and visualized in Cytoscape. Each node represents an individual MS/MS feature, while edges indicate spectral similarity between metabolites. Node colors correspond to the extraction solvents: N-hexane (red), ethyl acetate (dark blue), and methanol (light blue). The molecular network consists of 506 nodes. Five metabolites were annotated based on library matches with GNPS and verified with manual database: (**1**) 1,3,6,8-Tetrahydroxy-2-(1-hydroxyhexyl)anthracene-9,10-dione; (**2**) 13-Docosenamide; (**3**) 5-hydroxy-6E,8Z,11Z,14Z,17Z-eicosapentaenoic acid; (**4**) Atractylenolide III; and (**5**) Pheophorbide A.

The LC-HRMS analysis detected 506 molecular features (nodes), which were then grouped into several molecular clusters based on their spectral similarities (Figure 4). Of the detected nodes, five metabolites were dereplicated through library matching on the GNPS platform and manual verification using databases (Table 1). These compounds represent several chemical classes, namely terpenoids (Atractylenolide III), alkaloids (Pheophorbide A), polyketides (1,3,6,8-Tetrahydroxy-2-(1-hydroxyhexyl)anthracene-9,10-dione), and fatty acids (5-hydroxy-6E,8Z,11Z,14Z,17Z-eicosapentaenoic acid and 13-Docosenamide).

**Table 1:** Dereplicated compounds identified from *S. ilicifolium* under different solvents and extraction methods.

Compound Name	Parent Mass	Adduct	Extraction Method	Solvent	Location	Group	Bioactivity
1,3,6,8-Tetrahydroxy-2-(1-hydroxyhexyl)anthracene-9,10-dione	354.364	M-H_2_O+H	ME ^1^—Fresh Sample	Ethyl Acetate	Sundak Beach	Polyketides	Antimicrobial [33] and Cytotoxic [34]
13-docosenamide	675.662	2M+H	Maceration and ME—Fresh Sample	N-Hexane, Ethyl Acetate, and Methanol	Nguyahan and Sundak Beach	Fatty acids	Antioxidant activity [35]
5-hydroxy-6E,8Z,11Z,14Z,17Z-eicosapentaenoic acid	318.294	M-H	ME—Fresh Sample	Ethyl Acetate and Methanol	Nguyahan Beach	Fatty acids	Antioxidant activity [36], Antidiabetic activity [37]
Atractylenolide III	250.082	[M+Na]^+^	ME—Fresh Sample	N-Hexane	Nguyahan Beach	Terpenoids	Antioxidant activity [38].
Pheophorbide A	593.262	M+H	Maceration and ME—Fresh Sample	N-Hexane, Ethyl Acetate, and Methanol	Nguyahan and Sundak Beach	Alkaloids	Antioxidant activity [39].

^1^ Microwave extraction (ME).

**Figure 5:**
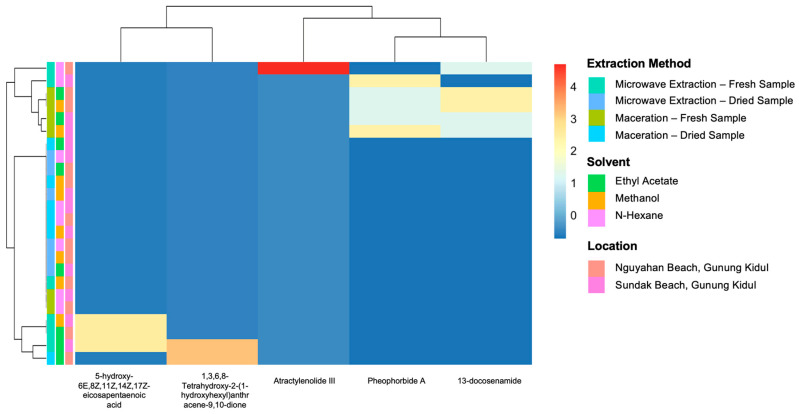
Heatmap of dereplicated metabolite abundance in *S. ilicifolium* extracts based on GNPS analysis. Samples were grouped based on extraction method (microwave extraction and maceration), solvent type (n-hexane, ethyl acetate, and methanol), sample condition (dry and fresh), and sampling location (Nguyahan Beach or Sundak Beach, Gunung Kidul), with metabolite intensity represented by a color scale from low (blue) to high (red).

To further explore the distribution patterns of identified metabolites across various extraction methods, solvent types, and sampling locations, heatmap analysis was performed based on the relative intensities obtained from GNPS (Figure 5).

**Figure 6:**
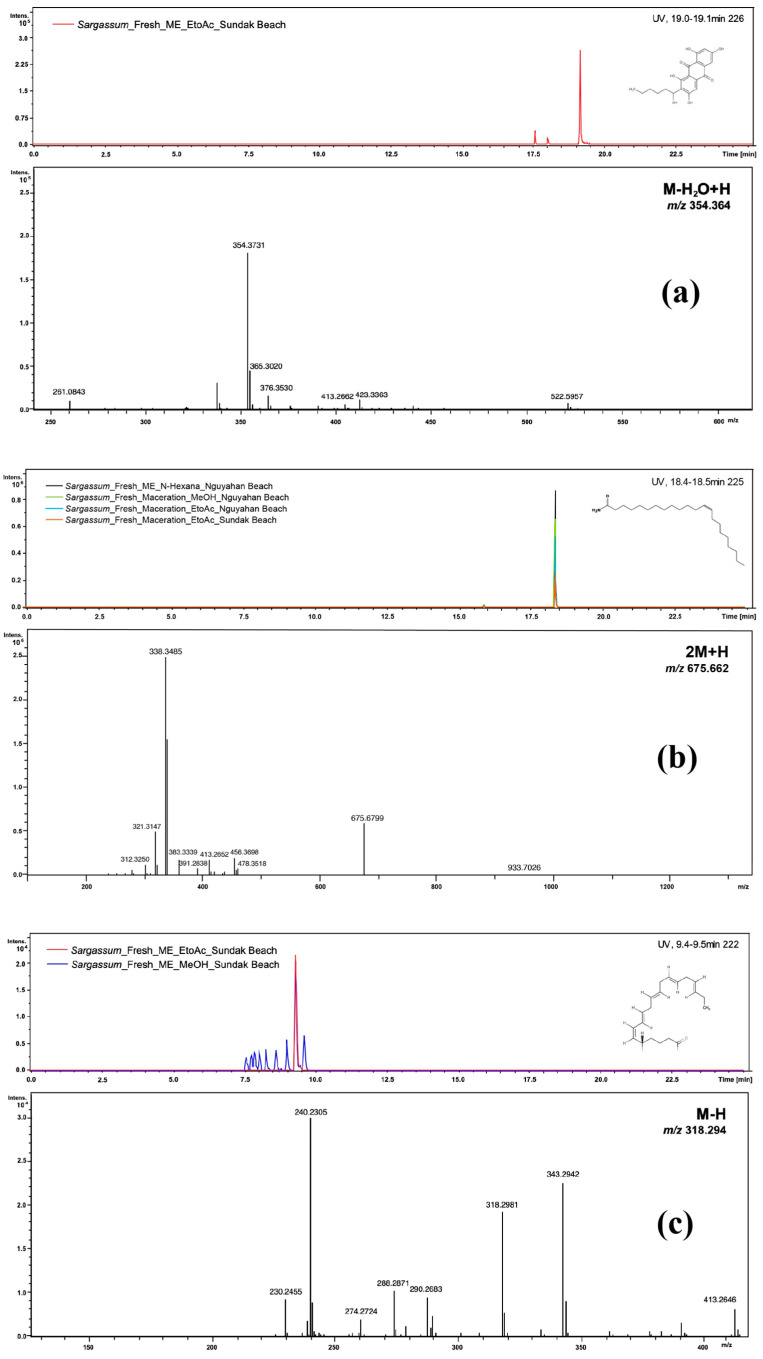

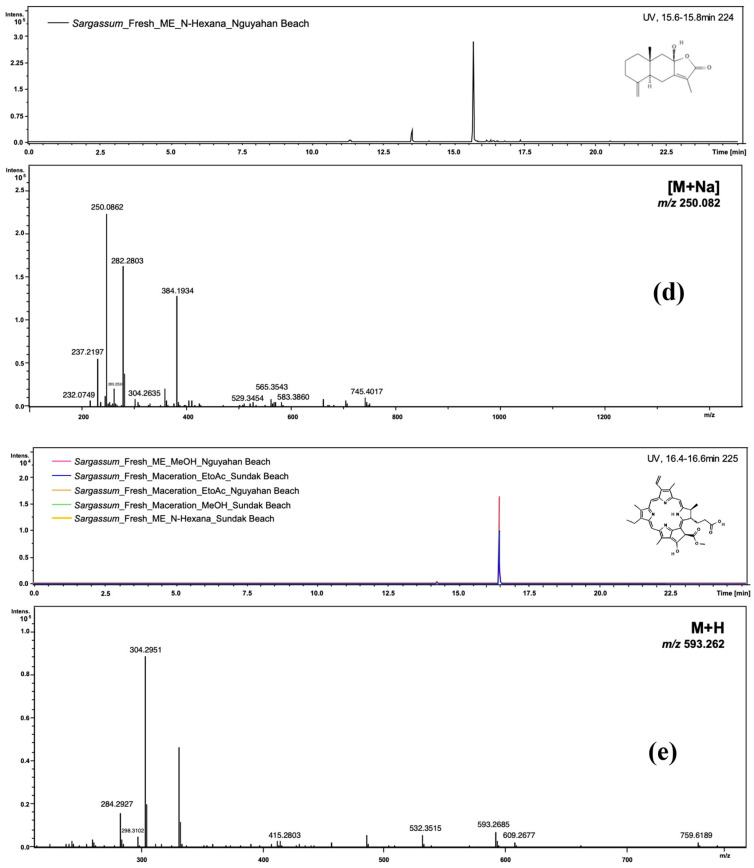
Extracted ion chromatograms (EICs) and MS/MS spectra of the identified compounds: (**a**) 1,3,6,8-Tetrahydroxy-2-(1-hydroxyhexyl)anthracene-9,10-dione, *m*/*z* 354.364 (M-H_2_O+H); (**b**) 13-Docosenamide, *m*/*z* 675.662 (2M+H); (**c**) 5-hydroxy-6E,8Z,11Z,14Z,17Z-eicosapentaenoic acid, *m*/*z* 318.294 (M-H); (**d**) Atractylenolide III, *m*/*z* 250.082 ([M+Na]^+^); (**e**) Pheophorbide A, *m*/*z* 593.262 (M+H).

Most of the dereplicated compounds were generated from fresh samples and extracted by the ME method. Compounds such as 13-docosenamide and pheophorbide A were found across nearly all solvent–method combinations, whereas atractylenolide III and 1,3,6,8-tetrahydroxy-2-(1-hydroxyhexyl)anthracene-9,10-dione appeared only under specific conditions (Figure 6).

### 3.2. Antioxidant Activity

**Figure 7:**
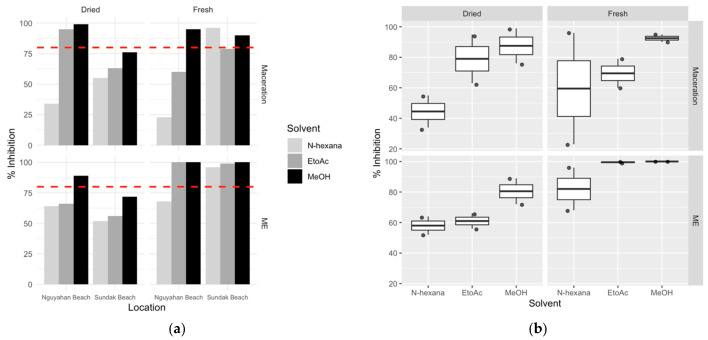
Antioxidant activity (% inhibition) of *S. ilicifolium* extract based on extraction method, solvent type, sample condition, and collection location. (**a**) Bar chart showing the percentage inhibition of dried and fresh samples from Nguyahan Beach and Sundak Beach using maceration and microwave-assisted extraction (ME) methods. Different colors indicate the solvent type: n-hexane (light gray), ethyl acetate (gray), and methanol (black). The dashed red line indicates the limit of strong antioxidant activity (≥80% inhibition). (**b**) Boxplot diagram showing the distribution of % inhibition values for each solvent under various conditions (dry vs. fresh; maceration vs. ME). Each box shows the interquartile range, the line within the box shows the median, and the dots indicate individual data points.

The antioxidant activity of *S. ilicifolium* extracts was evaluated using the DPPH radical scavenging assay. The percentage of antioxidant inhibition varies depending on sampling location, pretreatments, polarity of solvent, and extraction method (Figure 7).

**Figure 8:**
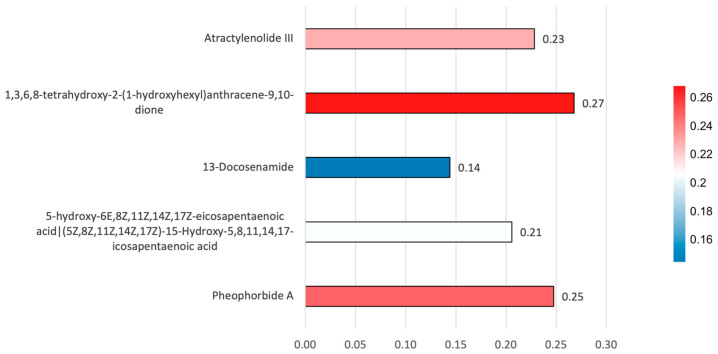
Correlation heatmap between identified metabolites and antioxidant inhibition values. Numbers within the cells represent Pearson correlation coefficients (r). The strength of the correlation is indicated by the color intensity: strong correlation (red); weak correlation (blue).

In general, antioxidant inhibition values differ between solvents. Methanol extracts tend to exhibit higher inhibition percentages compared to n-hexane and ethyl acetate, although these values vary slightly depending on the sampling location and sample conditions. In both maceration and microwave extraction (ME) methods, methanol extracts exhibited inhibition values in the relatively high range (around 70–100%). Meanwhile, ethyl acetate was in the intermediate range, and n-hexane consistently exhibited lower values (around 30–60%). Prior to statistical analysis, antioxidant inhibition values were tested to ensure that the parametric analysis assumptions were met. The Shapiro–Wilk normality test showed that all solvent groups had a normal data distribution (*p* > 0.05). Levene’s test also showed that the variance between groups was homogeneous (*p* = 0.115). The results of one-way ANOVA analysis showed that the type of solvent significantly affected the percentage of antioxidant inhibition (F(2,21) = 4.466; *p* = 0.024). Analysis using the Tukey HSD test showed that the extract with methanol solvent had a higher inhibition value than the extract with n-hexane solvent (*p* = 0.018). However, the difference between methanol and ethyl acetate did not show statistical significance. A three-way ANOVA analysis considering solvent, extraction method, and pretreatment showed that solvent was the only factor with a significant main effect on the inhibition percentage (*p* = 0.034). In contrast, the extraction method, pretreatment, and the interaction between these factors did not show a statistically significant effect (*p* > 0.05). To further explore the relationship between metabolite profiles and antioxidant activity, correlation analysis was performed to further determine the potential contribution of individual metabolites to antioxidant activity (Figure 8).

Correlation analysis showed a relationship between the detected metabolites and antioxidant inhibition values, although the strength of the correlation varied between compounds. The highest correlation was observed for 1,3,6,8-tetrahydroxy-2-(1-hydroxyhexyl)anthracene-9,10-dione (r = 0.27), followed by pheophorbide A (r = 0.25) and atractylenolide III (r = 0.23). A moderate correlation was also observed for 5-hydroxy-6E,8Z,11Z,14Z,17Z-eicosapentaenoic acid (r = 0.21), while 13-docosenamide showed the lowest correlation with DPPH inhibition (r = 0.14).

## 4. Discussion

### 4.1. Variation in Metabolite Profiles of Sargassum ilicifolium

Metabolite analysis showed that extraction conditions, particularly solvent polarity and the specific extraction method, play a significant role in metabolite recovery. For example, polar solvents such as methanol tend to extract more phenolic compounds, fucoidan, and sulfated polysaccharides. Non-polar solvents such as n-hexane tend to extract lipids, sterols, and pigments. The microwave extraction (ME) showed higher extraction efficiency compared to maceration. This efficiency is likely due to the effect of increased temperature and disruption of cell structure, which facilitates the release of metabolites into the solvent. However, high temperatures during the ME process can also cause the degradation of thermolabile compounds such as oxylipins and phyllobilins, as reported in previous studies of brown seaweeds. For example, the extraction of *Saccharina latissima* at a temperature of 120 °C is known to reduce the total phenolic and phlorotannin content [40]. Meanwhile, for *Sargassum filipendula*, excessively high temperatures can cause degradation of photosynthetic pigments [41]. This research shows that the extraction method used, especially the temperature in the extraction process, has an influence on the type of metabolite produced.

On the other hand, certain compounds such as atractylenolide III and 1,3,6,8-tetrahydroxy-2-(1-hydroxyhexyl)anthracene-9,10-dione were only found under ME conditions and nonpolar solvents. This indicates that these compounds are highly dependent on the type of solvent and extraction temperature. Therefore, the extraction method and type of solvent used are important in influencing the type and quantity of metabolites obtained from *S. ilicifolium* and, consequently, the potential bioactivity of the extract. Cluster analysis, as shown in the heatmap, indicates that differences in metabolite composition were influenced by diverse extraction conditions. Sample clustering is more influenced by the extraction method and solvent type than the sampling location. This suggests that the extraction method plays a more significant role in defining the metabolite composition of the *S. ilicifolium* extract than the geographic location in the sampling area.

### 4.2. Correlation Between Metabolites and Antioxidant Activity

The analysis of antioxidants revealed a pattern that was consistent with the data of our metabolomics analysis. The methanol extract tends to extract a greater amount of more polar compounds and demonstrates the highest activity on DPPH radical scavenging. Furthermore, the type of solvent used was found to significantly impact the antioxidant activity (*p* = 0.034). This suggests that the polarity of the solvent affects both the extracted compounds and their antioxidant activity. Each metabolite showed different metabolic pathways regarding the antioxidant activity. Atractylenolide III was shown to alter oxidative stress through suppression of intracellular reactive oxygen species (ROS) and also supports the endogenous antioxidant system by increasing the SOD-1 and HO-1 through the Nrf2 pathway [38]. Pheophorbide A also exhibits antioxidant activity by inhibition of ROS and alteration of the metabolism of several biological systems [39]. In contrast, several dereplicated compounds lack direct evidence of radical-scavenging activity.

The 13-docosenamide has primarily been associated with antimicrobial and cytotoxic properties [35] while its contribution to antioxidant activity is likely indirect. The compound 1,3,6,8-tetrahydroxy-2-(1-hydroxyhexyl)anthracene-9,10-dione, also known as Averantin (PubChem CID: 13945577), which is particularly produced from marine algal has not been previously reported as an antioxidant compound. Previous studies mainly describe its cytotoxic and antimicrobial effects [33,34]. In addition, 5-hydroxy-6E,8Z,11Z,14Z,17Z-eicosapentaenoic acid (5-HEPE), an EPA-derived oxylipin, is known to function as a lipid mediator involved in immune and inflammatory regulation rather than direct radical scavenging [42]. To further investigate the potential contribution of individual metabolites to the observed antioxidant activity, a correlation analysis between metabolite abundance and DPPH inhibition values was performed. The resulting correlations were generally positive but relatively weak (r = 0.14–0.27), indicating that none of the detected metabolites alone strongly explained the variation in antioxidant activity across samples. Among the detected metabolites, Atractylenolide III and pheophorbide A also showed moderate positive correlations with DPPH inhibition. Previous studies have reported that atractylenolide III is known to be associated with antioxidant defense mechanisms in cells, including activation of the Nrf2 signaling pathway [38]. Meanwhile, pheophorbide A is also known to influence oxidative processes by regulating ROS (reactive oxygen species) [39]. This suggests that these two compounds likely also contribute to the antioxidant activity of the extract.

In contrast, 13-docosenamide showed the lowest correlation with antioxidant inhibition values. This is likely due to its mechanism of activity, which does not directly act as a free radical scavenger. This compound is better known to have other biological activities, such as antimicrobial or cytotoxic [35], so its contribution to antioxidant activity as measured by the DPPH method is likely limited. Based on the DPPH test, antioxidant activity resulting from the extract *S. ilicifolium* is likely not derived from a single primary metabolite. This activity is likely the result of a combination of various metabolite groups. Some may act directly as antioxidants, while others may indirectly influence oxidative stress processes. This is consistent with reports in several studies of seaweed extracts, which show that biological activity is often influenced by the interaction of several compounds within a single extract [43,44]. Identification of bioactive metabolites in *S. ilicifolium* has the potential to be a source of compounds associated with antioxidant activity. Furthermore, the presence of various metabolite classes in the extract with high DPPH radical scavenging activity suggests the possibility of a combined effect between the compounds [45,46]. This study also shows that solvent polarity plays an important role in influencing the antioxidant inhibition values obtained from the samples. Methanol, which is the most polar solvent used in this study, generally produces a higher percentage of inhibition than ethyl acetate and n-hexane, although the degree of difference varied across locations and sample conditions. This pattern is consistent with the solubility properties of phenolic compounds and other polar antioxidant compounds in macroalgae, which tend to be more readily extracted using more polar organic solvents [43,44]. In contrast, n-hexane, as a nonpolar solvent, consistently produced the lowest inhibition values. Although there were slight variations in the influence of solvents at different locations, especially at Nguyahan Beach and Sundak Beach, the pattern of influence remained stable. The results of the statistical analysis even supported this. The data met the assumptions of normality and homogeneity of variance, allowing a valid ANOVA analysis to be performed. One-way ANOVA analysis showed that the type of solvent used was one of the variables determining the percentage of inhibition. On the other hand, through Tukey’s HSD test, the methanol extract showed greater antioxidant activity than the n-hexane extract. This was also supported by a three-way ANOVA, as the solvent was the only significant factor. At the same time, the extraction method (maceration and microwave extraction) and pretreatment did not show significant differences or interactions. This study revealed that the differences in antioxidant inhibition in these values were the result of the liquid solute and not due to the method used to extract the samples or the sample conditions.

Metabolite identification in this study is still tentative as it is based on spectrum matching through the Global Natural Products Social Molecular Networking (GNPS) platform. Additionally, antioxidant activity was assessed through only one in vitro assay method, DPPH, which measures free radical scavenging, but does not fully capture the mechanism of antioxidant activity. Antioxidant activity was expressed as a percentage inhibition to observe and compare the differences between extracts. It is suggested that future studies should focus on additional approaches to antioxidant activity, including specific activity per mg of extract, in order to evaluate antioxidant potential and to clarify the metabolite profile to biological activity relationship. This study confirms the relationship between extraction parameters, diversity of metabolites, and antioxidant capacity in *S. ilicifolium.* The antioxidant activity and diversity of metabolites extracted were largely dependent on the solvent’s polarity. This illustrates the necessity of selecting a suitable extraction method to fit the aims of the study, especially in untargeted metabolomics, where the metabolomics bioprofiling is integrated.

## 5. Conclusions

The combination of LC–HRMS-based metabolomics profiling and antioxidant activity provided a deeper understanding of the metabolite composition in *S. ilicifolium* collected from Nguyahan and Sundak Beaches, Gunungkidul, Indonesia. Molecular networking analysis identified 506 nodes, with five dereplicated compounds. These compounds included 13-docosenamide, atractylenolide III, pheophorbide A, 1,3,6,8-tetrahydroxy-2-(1-hydroxyhexyl)anthracene-9,10-dione, and 5-hydroxy-6E,8Z,11Z,14Z,17Z-eicosapentaenoic acid. Extraction method and solvent choice were found to influence the metabolite profile and antioxidant inhibition values. The methanolic *S. ilicifolium* extracts showed greater yields with the increased antioxidant inhibition activity compared to other solvents. Statistical analysis showed that the type of solvent was the main factor affecting the antioxidant inhibition values. The correlation between metabolomics data and antioxidant assay suggests that antioxidant activity is likely more influenced by contributions of several compounds, such as phenolic compounds, oxylipins, and chlorophyll derivatives, rather than a single dominant compound. This study emphasizes the importance of selecting extraction methods in metabolomics-based bioactivity studies and demonstrates the potential of *S. ilicifolium* as a source of bioactive secondary metabolites. This study provides a basis for further studies involving more targeted metabolite quantification, the use of various bioactivity assay methods, and further exploration of the pharmacological potential of *S. ilicifolium* extracts.

## Data Availability

The original contributions presented in this study are included in the article. Further inquiries can be directed to the corresponding authors.
